# Assessing measurement invariance in the EORTC QLQ-C30

**DOI:** 10.1007/s11136-021-02961-8

**Published:** 2021-07-29

**Authors:** Janneke van Roij, Jacobien M. Kieffer, Lonneke van de Poll-Franse, Olga Husson, Natasja J. H. Raijmakers, John Gelissen

**Affiliations:** 1 The Netherlands Comprehensive Cancer Organisation, PO box 19079, Utrecht, 3501 DB The Netherlands; 2 Department of Medical and Clinical Psychology, Center of Research on Psychology in Somatic Diseases (CoRPS), Tilburg University, Tilburg, the Netherlands; 3 Netherlands Association for Palliative Care (PZNL), Utrecht, The Netherlands; 4 Division of Psychosocial Research and Epidemiology, The Netherlands Cancer Institute, Amsterdam, The Netherlands; 5 The Institute of Cancer Research and the Royal Marsden NHS Foundation Trust, London, UK; 6 Department of Methodology and Statistics, Tilburg School of Social and Behavioral Sciences, Tilburg University, Tilburg, The Netherlands

**Keywords:** Measurement invariance, Quality of life, Medical oncology, Palliative care, Validation studies, Patient-reported outcomes

## Abstract

**Purpose:**

We aimed to investigate measurement invariance (MI) in the European Organisation for research and treatment of cancer quality of life questionnaire core 30 (EORTC QLQ-C30) in a heterogeneous sample of patients with cancer.

**Methods:**

Data from 12 studies within the PROFILES registry were used for secondary analyses (*n* = 7007). We tested MI by successive restrictions on thresholds, loadings, and intercepts across subgroups based on primary cancer sites, age, sex, time since diagnosis, and life stage, using multigroup confirmatory factor analysis (MGCFA) for ordered categorical measures. We also evaluated the impact of potentially miss-specified parameter equality across groups on latent factor means by releasing threshold and loading equality constraints for each item at a time.

**Results:**

Results showed that the highest level of MI (invariance of thresholds, loadings, and intercepts) was found across groups based on time since diagnosis and life stage and to a lesser extent across groups based on sex, age, and primary tumor site. On item level, however, changes in the item’s associated factor means were relatively small and in most cases canceled each other out to some extent.

**Conclusions:**

Given only a few instances of non-invariance in our study, there is reason to be confident that valid conclusions can be drawn from between-group comparisons of QLQ-C30 latent means as operationalized in our study. Nonetheless, further research into MI between other subgroups for the QLQ-C30 (i.e., treatment effects and ethnicity) is warranted. We stress the importance of including MI evaluations in the development and validation of measurement instruments.

**Supplementary Information:**

The online version contains supplementary material available at 10.1007/s11136-021-02961-8.

## Introduction

An important measurement property of a questionnaire is measurement invariance (MI), which states that the relationship between the items of a questionnaire and the latent construct that is measured is stable and independent of group membership or the measurement occasion [1]. If the assumption of MI is violated, observed differences between groups are not true differences in the construct of interest but may reflect systematic error. In other words, when using a questionnaire for group comparison, members of different groups must assign the same meaning to the items and scale that indicate the construct. Only when a questionnaire is MI, a valid comparison can be made between groups [2].

One of the most widely used quality of life (QoL) questionnaires in cancer research is the European Organisation for Research and Treatment of Cancer Quality of Life Questionnaire Core 30 (EORTC QLQ-C30) [3]. Only a few studies have examined the MI of the EORTC QLQ-C30 concerning change over time, and clinical and patient characteristics. A small qualitative study in a heterogeneous cancer sample showed different cognitive processes underlying QoL appraisal before and after radiotherapy in patients with cancer [4]. In prostate cancer patients, the physical functioning and role functioning subscales of the QLQ-C30 gained importance over time for representing the QoL construct. The same study showed that a change in internal standards (a form of response shift) made patients perceive their emotional and cognitive functioning more positive at follow-up [5]. In a larger study (*n* > 30000) with a multicultural heterogeneous cancer sample, however, researchers found little measurement bias in the QLQ-C30 across time points of assessment (baseline, on-treatment, and off-treatment), regardless of treatment status [6].

Concerning other clinical and patient characteristics, one study showed measurement bias for age and previous treatment in the QLQ-C30, but not for sex and treatment preference [7]. However, the sample size of this study was rather small, considering the statistical approach used. In contrast, the QLQ-C30 was found to be MI for age, sex, and type of surgery (i.e., robot- or not robot-assisted) in lung cancer patients [8], and for primary cancer sites in a large heterogeneous cancer sample (*n* = 1906) [9]. Two other studies found that the QLQ-C30 was mostly MI across ethnic groups [10], languages [11], countries [12].

The QLQ-C30 is designed to measure QoL in heterogeneous cancer populations, which could lead to measurement non-invariance and biased group comparisons. Because the literature on MI of this questionnaire is limited, we investigate the MI of the QLQ-C30 in a large Dutch patient sample with different primary cancer sites. Additionally, potential measurement bias concerning age, sex, time since diagnosis, and life stage (i.e., cancer survivors versus patients in their last year of life) is evaluated.

## Methods

### Data source

Data from the PROFILES ‘(Patient-Reported Outcomes Following Initial treatment and Long term Evaluation of Survivorship)’ registry were used for secondary analyses. The PROFILES registry (www.profilesregistry.nl) is an ongoing collection of patient-reported outcomes from studies on various cancer types, within the sampling frame of the Netherlands Cancer Registry (NCR), and can be linked with clinical data of all individuals newly diagnosed with cancer in the Netherlands (see [13] for a detailed description of the data collection for the PROFILES registry).

### Study population

The current analysis is based on data from 12 studies from the PROFILES registry, including 7460 patients, of whom approximately 300 patients are in their last year of life (i.e., patients with cancer who died within one year after completing the questionnaire). Patients were included in studies between May 2009 and October 2015. Study samples varied by size, inclusion criteria, and primary cancer site. Participants were included if they were older than 18 years and excluded if they were not able to complete a Dutch questionnaire (i.e., cognitive impairment, non-native speaker, too ill to participate). Ethical approval was obtained for all studies separately from a local certified medical ethics committee.

Socio-demographic and clinical data were obtained from the NCR. Socio-demographic variables included age, sex, educational level, and relationship status. Age groups (18–44, 45–65, > 65 years old) were created based on a minimum number of patients of 400 in each age category. Clinical data included comorbidity, primary cancer site, and date of primary diagnosis. Comorbidity at time of survey was classified according to the adapted Self-administered Comorbidity Questionnaire (SCQ) [14] and categorized into no physical comorbidities, one or > 1 physical comorbidities. Primary cancer site was classified according to the third International Classification of Diseases for Oncology (ICDO-3) [15]. Primary cancer sites included in this study are colorectal cancer, prostate cancer, ovarian cancer, endometrial cancer, melanoma, thyroid cancer, Hodgkin lymphoma, non-Hodgkin lymphoma, multiple myeloma, chronic lymphocytic leukemia, and basal cell/squamous cell carcinoma. Dates of death of patients were obtained from the Dutch municipal personal records database and were last verified on February 1st, 2017.

### The EORTC QLQ-C30

The 30-item EORTC QLQ-C30 is a disease-specific measure that assesses multiple QoL domains in patients with cancer. There are five functioning scales that measure physical, role, emotional, cognitive, and social functioning. Three symptom scales measure fatigue, pain, and nausea/vomiting. One scale assesses global health and QoL. The questionnaire includes six single items assessing cancer-related problems (i.e., dyspnea, sleep problems, appetite loss, constipation, diarrhea, and financial difficulties). Responses range on a four-point scale from 1 ‘Not at all’ to 4 ‘Very much’, except for the global QoL scale items, which have a 7-point response format from 1 ‘Very poor’ to 7 ‘Excellent’. For the functioning and global QoL scale, a higher score indicates better health. For symptoms scales, a higher score indicates a higher level of symptom burden [3].

### Statistical analysis

Scores on the QLQ-C30 were calculated according to published scoring algorithms. As our data was not missing completely at random (as indicated by Little’s MCAR test), associations between socio-demographic variables and missingness in our data were explored. Higher educated patients were relatively less likely to generate missing data compared to patients with lower levels of education. Patients with a partner were for some indicators less likely, and patients in their last year of life were more likely to generate missing data. In logistic regressions for missingness on the indicators, the dependent variables (missing or not for each item) were in almost all instances highly skewed and the large sample size made these findings only indicative. Following EORTC guidelines, missing values were replaced by the average score of the completed items in the same scale for each individual, provided that at least 50% of the items in that scale had been completed [16]. Of the total of 7460 cases in our sample, 6636 were initially complete (89%); after imputation according to the EORTQ guidelines, we had 7007 (94%) complete cases. A *p*-value < 0.05 was set to be statistically significant for all analyses.

**Fig. 1:**
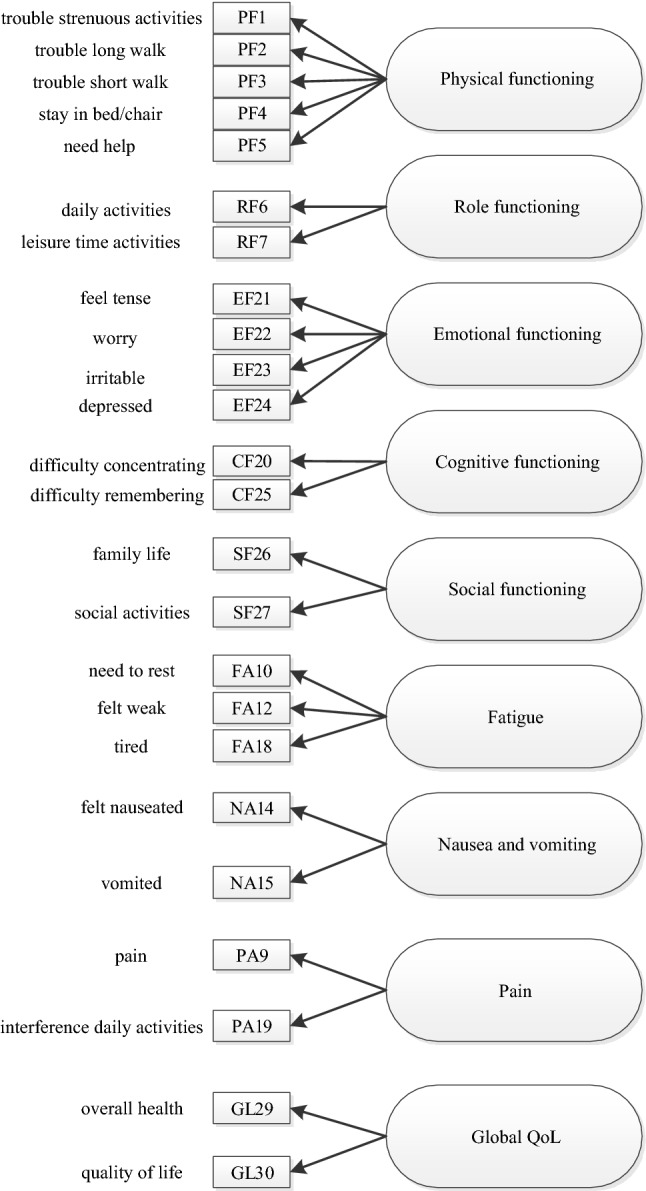
Measurement model of European Organisation for research and treatment of cancer quality of life questionnaire core 30 (EORTC QLQ-C30)

Single-group confirmatory factor analysis with ordered categorical indicators was used first to evaluate the appropriateness of the original QLQ-C30 model in the separate subpopulations of the grouping variables: primary cancer sites, age, sex, time since diagnosis, and life stage. This analysis was followed by multigroup confirmatory factor analysis (MGCFA) with ordered categorical measures to evaluate MI of the original measurement model of the QLQ-C30 across the various grouping variables. For model identification purposes we only included scales of the QLQ-C30 with at least two indicators (Fig. 1). All analysis were done using the lavaan package in R [17] and the measEq.syntax function within the semTools package [18]. Because nearly all indicators in this study were ordered categorical, we tested the multiple-groups invariance of constructs following the model identification approach of Wu and Estabrook [19] and as laid out in detailed guidelines by Svetina, Rutkowski, and Rutkowski [20]. Specifically, we used the diagonally weighted least squares estimator (DWLS) with the mean- and variance adjustment procedure [21] and with the delta parametrization [22]. To assess the degree to which the independence-of-observations assumption may have been violated, we estimated Intraclass correlation Coefficients (ICCs) with multilevel mixed-effects ordered logistic models for each ordinal indicator and ‘study’ as the cluster variable. The clustering effects were very small, with the average ICC across all indicators being 0.04 (SD = 0.02). For all SEM models, we used pairwise deletion of missing values.

MI was explored through a sequence of steps appropriate for latent variable models with ordered categorical indicators [19], starting with a successive implementation of restrictions on the model parameters: configural model, in this model all parameters are freely estimated to test if the same pattern of loadings can be applied in each group. Model with equal thresholds: suggesting similar observed proportions for each response category, implied by thresholds in the latent distribution. Model with equal thresholds and equal loadings, indicating same meaning of latent construct; We estimated models in which thresholds and factor loadings were constrained to be equal across groups, but the item intercepts were allowed to differ between groups. Model with equal thresholds, factor loadings, and intercepts, indicating same meaning and scale of the construct. We estimated models in which the thresholds and factor loadings are constrained to be equal and the levels of the underlying items (intercepts) are equal in both groups; the groups are comparable on their subscale scores.

The Satorra–Bentler scaled (mean-adjusted) Chi-square was calculated for each model. This is the standard (normal-theory) Chi-square statistic divided by a scaling correction to better approximate a Chi-square under non-normality. MI models were compared using a Chi-square difference test for the Satorra–Bentler scaled Chi-square. A significant Chi-square difference (∆*χ*^2^) test would indicate worse fit of the more constrained model compared to the less constrained model [23]. We also reported the root mean square error of approximation (RMSEA), where values < 0.06 indicate good fit, and < 0.08 acceptable), the comparative fit index (CFI), and the Tucker–Lewis index (TLI), for both values > 0.95 indicate good fit [2, 24]. Because these goodness-of-fit statistics are derived from the models using the Chi-squared test, they too are scaled and become robust to non-normality [25].

As Chi-square tests are sensitive to large sample sizes, we evaluated the impact of potentially miss-specified parameter equality across groups on latent factor means by releasing threshold and loading equality constraints for each item at a time, with intercept and residual variance fixed to 0 and 1, respectively [26]. We tested the partial invariant models (depending on the groups compared) against the model with equal intercepts using scaled χ2 difference tests and we reported the changes in the deviations between-group-specific latent variable means (reference group vs. Comparison groups). A change in latent factor estimates < 0.2 indicated that the impact of parameter equality across groups on latent factor means was small, and changes of 0.40 and 0.70 indicated medium, respectively large effects [27].

## Results

In total, 7007 patients with cancer were included in the analysis, of which 266 patients were in their last year of life (Table 1). Patients in the total sample were often male (58%) and were on average 66 years old (SD = 12). The majority of the patients (69%) were diagnosed with a primary tumor less than five years ago and 68% had physical comorbidities. Patients in their last year of life were on average older (71, SD = 9) and more often had physical comorbidities (75%) compared to patients in the total sample. Ovarian cancer and multiple myeloma was relatively more prevalent in patients in their last year of life (11 and 12% respectively) compared to the total sample (5 and 4% respectively).

**Table 1:** Socio-demographics and clinical characteristics study population

	Total sample (*n* = 7007) (*n*) %	Cancer survivors (*n* = 6741) (*n*) %	Patients with cancer in their last year of life* (*n* = 266) (*n*) %
Sex			
Male	58 (4082)	58 (3925)	59 (157)
Age			
Mean (sd), range	66 (12), 18–97	66 (12), 18–97	71 (9), 40–96
18–44 years	6 (405)	6 (403)	1 (2)
45–65 years	36 (2516)	36 (2444)	27 (72)
> 65 years	58 (4064)	58 (3872)	72 (192)
Relationship status			
Partner	78 (5414)	78 (5228)	71 (186)
No partner	22 (1526)	22 (1450)	29 (76)
Education			
Lower education or less	16 (1141)	16 (1092)	19 (49)
Secondary education (high school, vocational)	62 (4261)	62 (4098)	63 (163)
University, higher (vocational) education	22 (1519)	22 (1471)	18 (48)
Primary cancer site			
Colorectal cancer	35 (2444)	35 (2,349)	36 (95)
Prostate cancer	16 (1104)	16 (1,076)	11 (28)
Non-Hodgkin lymphoma	15 (1073)	15 (1,027)	17 (46)
Basal cell/squamous cell carcinoma	9 (657)	10 (649)	3 (8)
Ovarian cancer	5 (342)	5 (311)	12 (31)
Thyroid cancer	4 (297)	4 (290)	1 (2)
Chronic lymphocytic leukemia	4 (277)	4 (262)	6 (15)
Multiple myeloma	3 (242)	3 (208)	13 (34)
Melanoma	3 (225)	3 (223)	1 (2)
Endometrial cancer	2 (142)	2 (139)	1 (3)
Hodgkin lymphoma	3 (209)	3 (207)	1 (2)
Time since diagnosis			
Mean (sd), range	4 (3), 0–21	4 (3), 0–21	3 (3), 0–19
0–2 years	27 (1873)	26 (1785)	33 (88)
3–5 years	42 (2957)	42 (2831)	47 (126)
> 5 years	31 (2177)	32 (2125)	20 (52)
Physical comorbidities			
Yes	68 (4774)	68 (4574)	75 (200)
No	32 (2233)	32 (2167)	25 (66)

Missings < 3% are not shown, physical comorbidities are self-reported physical comorbid conditions present in the last 12 months, e.g., heart condition, stroke, high blood pressure, asthma, chronic bronchitis, COPD, diabetes, ulcer, kidney disease, liver disease, anemia or other blood condition, thyroid disease, arthritis, backache, or rheumatism

*sd* Standard deviation

*Patients with cancer who died within one year after completing the questionnaire

### Primary cancer site-related MI

As we found negative error variances for multiple items across primary cancer sites, we decided to compare sites with positive error variances patterns in the items and to exclude the related construct that contained an item with a negative error variance from the group comparison. If necessary, we also collapsed response categories when there were zero observations in one of the categories of an ordinal indicator. For the comparison of colorectal and Hodgkin lymphoma, response categories of items PF5 (Help eating, dressing, washing, using the toilet) and NA15 (Vomiting) were recoded to three. Due to negative error variances the Pain construct was excluded from the model in the comparison of multiple myeloma and basal cell/squamous cell carcinoma, and the Nausea construct was omitted in the comparison of prostate cancer, thyroid cancer, non-Hodgkin lymphoma, and chronic lymphocytic leukemia. In the latter comparison, the response categories of item PF4 were collapsed to three. We decided to exclude the primary cancer sites ovarian, uterine, and melanoma cancer from our analysis, as we found negative error variances on multiple constructs.

Fit indices showed good fit (CFI and TLI > 0.95 and RMSEA < 0.06) for the measurement model in the separate primary cancer sites (Table 2). All invariance models (Table 3) fitted the data well (CFI and TLI > 0.95 and RMSEA < 0.06). Colorectal cancer, ovarian cancer, and Hodgkin lymphoma reached the highest level of MI (∆*χ*^2^ *p* > 0.05), indicating invariance of the thresholds, factor loadings, and intercepts. In the comparison of multiple myeloma with basal cell/squamous cell carcinoma, and in the comparison of prostate cancer with thyroid cancer, Non-Hodgkin lymphoma, and chronic lymphocytic leukemia Chi-square difference tests were significant between the model with equal thresholds and the model with equal loadings (∆*χ*^2^ *p* < 0.05) indicating invariance of thresholds.

**Table 2:** Factor loading estimates of single group models on EORTC QLQ-C30 scales

Items	Primary cancer site								
	Colorectal cancer	Prostate cancer	Ovarian cancer	Non-Hodgkin lymphoma	Multiple myeloma	Basal cell/squamous cell carcinoma	Chronic lymphocytic leukemia	Thyroid cancer	Hodgkin lymphoma
Physical functioning									
Q1	1	1	1	1	1	1	1	1	1
Q2	1.030 (0.013)	1.003 (0.017)	1.051 (0.026)	1.029 (0.017)	1.031 (0.038)	1.098 (0.024)	0.923 (0.027)	0.984 (0.028)	1.036 (0.057)
Q3	1.047 (.013)	1.022 (0.019)	0.996 (0.028)	1.020 (0.017)	1.025 (0.040)	1.098 (0.019)	0.941 (0.031)	0.999 (0.037)	1.037 (0.068)
Q4	0.989 (0.025)	0.984 (0.035)	0.977 (0.038)	0.923 (0.033)	1.000 (0.047)	0.989 (0.045)	0.931 (0.047)	0.824 (0.068)	1.115 (0.103)
Q5	0.839 (0.037)	0.950 (0.047)	0.807 (0.088)	0.848 (0.058)	1.048 (0.067)	0.870 (0.069)	0.672 (0.120)	0.782 (0.121)	1.025 (0.216)
Role functioning									
Q6	1	1	1	1	1	1	1	1	1
Q7	0.949 (0.010)	0.969 (0.011)	0.927 (0.023)	0.983 (0.013)	0.973 (0.018)	0.965 (0.023)	0.969 (0.023)	0.944 0 (0.023)	0.987 (0.027)
Emotional functioning									
Q21	1	1	1	1	1	1	1	1	1
Q22	0.988 (0.014)	0.946 (0.019)	1.022 (0.038)	0.978 (0.021)	1.017 (0.042)	0.924 (0.023)	0.967 (0.025)	1.023 (0.048)	0.966 (0.035)
Q23	0.948 (0.014)	0.951 (0.016)	0.954 (0.042)	0.958 (0.019)	0.939 (0.040)	0.938 (0.021)	0.976 (0.026)	0.995 (0.044)	1.007 (0.026)
Q24	1.018 (0.012)	0.987 (0.018)	1.076 (0.037)	0.979 (0.020)	1.078 (0.036)	0.948 (0.023)	1.003 (0.024)	1.022 (0.043)	0.979 (0.034)
Cognitive functioning									
Q20	1	1	1	1	1	1	1	1	1
Q25	0.741 (0.025)	0.699 (0.035)	0.836 (0.093)	0.777 (0.032)	0.721 (0.075)	0.935 (0.044)	0.819 (0.055)	0.763 (0.067)	0.834 (0.069)
Social functioning									
Q26	1	1	–	1	1	1	1	1	1
Q27	1.115 (0.023)	1.154 (0.037)		1.144 (0.034)	1.115 (0.048)	0.993 (0.042)	1.133 (0.058)	1.152 (0.065)	1.110 (0.067)
Fatigue									
Q10	1	1	1	1	1	1	1	1	1
Q12	1.006 (0.013)	0.951 (0.016)	0.975 (0.024)	0.974 (0.017)	1.021 (0.032)	0.921 (0.023)	0.969 (0.024)	0.915 (0.032)	0.958 (0.030)
Q18	1.009 (0.012)	0.987 (0.015)	0.978 (0.026)	1.031 (0.017)	0.995 (0.034)	0.968 (0.019)	1.029 (0.020)	0.923 (0.026)	0.917 (0.031)
Nausea and vomiting									
Q14	1	-	1	–	1	1	–	–	1
Q15	0.911 (0.059)		0.922 (0.088)		1.040 (0.123)	0.906 (0.165)			0.942 (0.144)
Pain									
Q9	1	1	1	1	–	–	1	1	1
Q19	1.090 (0.018)	1.133 (0.026)	1.102 (0.046)	1.011 (0.022)			1.031 (0.039)	1.097 (0.045)	1.046 (0.045)
Global QoL									
Q29	1	1	1	1	1	1	1	1	1
Q30	0.961 (0.015)	0.888 (0.023)	0.897 (0.037)	0.955 (0.024)	1.014 (0.040)	0.946 (0.040)	1.031 (0.039)	0.917 (0.045)	0.921 (0.066)
Scaled *χ*^2^ (df) *p*-value	1459.677 (216), *p* < 0.001	547.615 (181), *p* < 0.001	267.545 (181), *p* < 0.001	580.964 (181), *p* < 0.001	313.889 (181), *p* < 0.001	346.826 (181), *p* < 0.001	250.287 (181), *p* < 0.001	267.645 (181), *p* < 0.001	258.940 (216), *p* = 0.024
Scaled RMSEA (90% CI)	0.047 (0.045–0.049)	0.042 (0.038–0.046)	0036 (0.027–0.045)	0.044 (0.040–0.048)	0.053 (0.043–0.063)	0.036 (0.031–0.042)	0.036 (0.024–0.047)	0.040 (0.029–0.050)	0.031 (0.012–0.044)
Scaled CFI	0.988	0.993	0.995	0.993	0.989	0.993	0.996	0.994	0.995
Scaled TLI	0.985	0.991	0.993	0.991	0.986	0.991	0.995	0.992	0.994

Items	Sex		Age			Time since diagnosis			Life stage	
	Male	Female	18–44 years	45–65 years	> 65 years	0–2 years	3–5 years	> 5 years	Cancer survivors	Patients with cancer in their last year of life*
Physical functioning										
Q1	1	1	1	1	1	1	1	1	1	1
Q2	1.009 (0.009)	1.046 (0.010)	1.073 (0.034)	1.026 (0.011)	1.027 (0.008)	1.037 (0.011)	1.024 (0.011)	1.012 (0.011)	1.027 (0.007)	1.027 (.028)
Q3	1.027 (0.010)	1.044 (0.010)	1.050 (0.042)	1.040 (0.012)	1.038 (0.009)	1.032 (0.011)	1.039 (0.012)	1.021 (0.012)	1.037 (0.007)	1.005 (.033)
Q4	0.972 (0.018)	0.973 (0.019)	1.074 (0.049)	0.955 (0.021)	0.962 (0.017)	0.981 (0.023)	0.986 (0.021)	0.924 (0.025)	0.953 (0.015)	1.038 (.036)
Q5	0.868 (0.030)	0.834 (0.032)	1.037 (0.162)	0.887 (0.041)	0.850 (0.025)	0.852 (0.039)	0.866 (0.036)	0.832 (0.037)	0.818 (0.024)	0.941 (0.058)
Role functioning										
Q6	1	1	1	1	1	1	1	1	1	1
Q7	0.979 (0.006)	0.955 (0.008)	1.003 (0.018)	0.975 (0.009)	0.953 (0.006)	0.976 (0.009)	0.968 (0.008)	0.945 (0.010)	0.960 (0.005)	0.985 (0.017)
Emotional functioning										
Q21	1	1	1	1	1	1	1	1	1	1
Q22	0.982 (0.010)	0.963 (0.012)	0.962 (0.030)	0.966 (0.012)	0.986 (0.011)	0.977 (0.015)	0.968 (0.011)	0.988 (0.013)	0.980 (0.008)	0.964 (0.040)
Q23	0.980 (0.009)	0.937 (0.011)	1.000 (0.028)	0.981 (0.011)	0.941 (0.010)	0.958 (0.015)	0.943 (0.011)	0.980 (0.012)	0.958 (0.007)	0.927 (0.036)
Q24	1.016 (0.010)	0.998 (0.011)	0.947 (0.030)	1.017 (0.011)	1.006 (0.010)	1.019 (0.015)	0.999 (0.010)	1.007 (0.012)	1.008 (0.007)	1.005 (0.037)
Cognitive functioning										
Q20	1	1	1	1	1	1	1	1	–	–
Q25	0.767 (0.017)	0.791 (0.022)	0.824 (0.047)	0.849 (0.020)	0.707 (0.019)	0.820 (0.024)	0.749 (0.021)	0.757 (0.025)		
Social functioning										
Q26	1	1	1	1	1	1	1	1	1	1
Q27	1.121 (0.017)	1.120 (0.020)	1.094 (0.047)	1.105 (0.018)	1.141 (0.019)	1.094 (0.021)	1.116 (0.019)	1.168 (0.027)	1.123 (0.013)	1.218 (0.068)
Fatigue										
Q10	1	1	1	1	1	1	1	1	1	1
Q12	0.985 (0.008)	0.959 (0.010)	0.925 (0.020)	0.962 (0.010)	0.979 (0.009)	0.951 (0.010)	0.990 (0.010)	0.957 (0.014)	0.962 (0.007)	1.009 (0.026)
Q18	0.999 (0.008)	0.984 (0.010)	0.934 (0.019)	0.983 (0.010)	1.015 (0.009)	0.983 (0.010)	1.007 (0.010)	0.988 (0.012)	0.990 (0.007)	1.066 (0.027)
Nausea and vomiting										
Q14	1	1	–	–	–	–	–	–	–	–
Q15	0.846 (0.051)	0.829 (0.045)								
Pain										
Q9	1	1	1	1	1	1	1	1	1	1
Q19	1.104 (0.014)	1.081 (0.013)	1.043 (0.041)	1.065 (0.016)	1.116 (0.013)	1.106 (0.018)	1.094 (0.015)	1.079 (0.016)	1.094 (0.010)	1.108 (0.055)
Global										
Q29	1	1	1	1	1	1	1	1	1	1
Q30	0.944 (0.012)	0.947 (0.014)	1.003 (0.046)	0.912 (0.015)	0.960 (0.011)	0.944 (0.016)	0.937 (0.014)	0.958 (0.017)	0.942 (0.009)	0.964 (0.050)
Scaled *χ*^2^ (df), *p*-value	2207.829 (216), *p* < 0.001	1609.921 (216), *p* < 0.001	224.623 (181), *p* = 0.015	955.555 (181), *p* < 0.001	2029.246 (181), *p* < 0.001	943.085 (181), *p* < 0.001	1509.818 (181), *p* < 0.001	1180.068 (181), *p* < 0.001	2788.004 (149), *p* < 0.001	264.984 (149), *p* < 0.001
Scaled RMSEA (90% CI)	0.046 (0.045–0.048)	0.045 (0.043–0.047)	0.024 (0.011–0.034)	0.040 (0.038–0.043)	0.048 (0.046–0.050)	0.046 (0.043–0.048)	0.049 (0.046–0.051)	0.049 (0.046–0.052)	0.050 (0.048–0.051)	0.051 (0.041–0.061)
Scaled CFI	0.990	0.990	0.998	0.994	0.990	0.993	0.991	0.990	0.991	0.994
Scaled TLI	0.987	0.987	0.997	0.993	0.988	0.991	0.988	0.987	0.989	0.992

*df* Degrees of freedom, *EORTC QLQ-C30* European Organisation for research and treatment of cancer quality of life questionnaire core 30, *Q* question, *RMSEA* root mean square error of approximation, *CI* confidence interval, *CFI* confirmatory fit index, *TLI* tucker lewis index

*Patients with cancer who died within one year after completing the questionnaire

**Table 3:** Fit indices and results of chi-squared difference tests of multiple-group models for testing configural invariance and successive invariance of thresholds, loadings and intercepts of EORTC QLQ-C30 scales

	Scaled *χ*^2^	df	p-value	Scaled RMSEA	Scaled CFI	Scaled TLI	Scaled *χ*^2^ difference test			
							Standard *χ*^2^	*χ*^2^ difference	df difference	*p*-value
Grouping variable: primary cancer site										
Colorectal cancer (*n* = 2444) and Hodgkin lymphoma (*n* = 209)										
Configural	1283.946	432	< 0.001	0.038	0.992	0.989	993.25	–	–	–
Equal thresholds	1282.974	452	< 0.001	0.036	0.992	0.990	1000.75	19.076	20	0.517
Equal loadings	1249.969	467	< 0.001	0.035	0.992	0.991	1012.9	15.429	15	0.421
Equal intercepts	1201.123	482	< 0.001	0.033	0.993	0.992	141.90	17.683	15	0.280
Multiple myeloma (*n* = 242) and basal cell/squamous cell carcinoma (*n* = 657)										
Configural	660.078	362	< 0.001	0.042	0.992	0.99	397.00	–	–	–
Equal thresholds	677.068	382	< 0.001	0.040	0.992	0.99	402.24	13.957	20	0.833
Equal loadings	702.565	396	< 0.001	0.040	0.992	0.99	427.20	30.763	14	0.006
Equal intercepts	747.542	410	< 0.001	0.042	0.991	0.99	488.6	44.033	14	< 0.001
Prostate cancer (*n* = 1104), thyroid cancer (*n* = 297), non-Hodgkin lymphoma (*n* = 1073), and chronic lymphocytic leukemia (*n* = 277)										
Configural	1615.123	724	< 0.001	0.041	0.994	0.992	932.95	–	–	–
Equal thresholds	1658.294	781	< 0.001	0.039	0.994	0.992	949.87	50.886	57	0.702
Equal loadings	1715.653	823	< 0.001	0.039	0.994	0.993	1007.77	60.539	42	0.032
Equal intercepts	1675.691	865	< 0.001	0.036	0.994	0.994	1090.1	50.592	42	0.171
Grouping variable: sex										
Male (*n* = 4082) and female (*n* = 2925)										
Configural	3771.668	432	< 0.001	0.046	0.990	0.987	2110.9	–	–	–
Equal thresholds	3830.818	454	< 0.001	0.045	0.990	0.988	2118.7	18.813	22	0.657
Equal loadings	3830.739	469	< 0.001	0.044	0.990	0.988	2149.1	39.097	15	0.001
Equal intercepts	3804.084	484	< 0.001	0.043	0.990	0.989	2340.4	116.95	15	< 0.001
Grouping variable: age										
18–44 years (*n* = 405), 45–65 years (*n* = 2516), and > 65 years (*n* = 4064)										
Configural	3096.996	543	< 0.001	0.044	0.989	0.986	1837.9	–	–	–
Equal thresholds	3161.510	583	< 0.001	0.042	0.989	0.987	1865.1	47.507	40	0.193
Equal loadings	3188.809	611	< 0.001	0.041	0.989	0.988	1960.4	54.828	28	0.002
Equal intercepts	3258.656	639	< 0.001	0.041	0.989	0.988	2182.4	79.250	28	< 0.001
Grouping variable: time since diagnosis										
0–2 years (*n* = 1873), 3–5 years (*n* = 2957), and > 5 years (*n* = 2177)										
Configural	3857.685	543	< 0.001	0.050	0.987	0.983	2099.4	–	–	–
Equal thresholds	3950.677	583	< 0.001	0.048	0.986	0.984	2124.3	51.048	40	0.113
Equal loadings	3892.166	611	< 0.001	0.047	0.987	0.985	2149.1	30.7	28	0.331
Equal intercepts	3736.463	639	< 0.001	0.044	0.987	0.986	2199.1	44.105	28	0.027
Grouping variable: life stage										
Cancer survivors (*n* = 6741) and patients with cancer in their last year of life (*n* = 266)*										
Configural	3102.375	298	< 0.001	0.050	0.987	0.983	1781.3	–	–	–
Equal thresholds	3130.363	316	< 0.001	0.049	0.987	0.984	1795.1	21.164	18	0.271
Equal loadings	3041.190	329	< 0.001	0.047	0.987	0.984	1806.9	13.639	13	0.400
Equal intercepts	3015.308	342	< 0.001	0.046	0.988	0.986	1842.1	21.217	13	0.069

Scaled *χ*^2^ and Scaled *χ*^2^ difference test with Satorra (2000) scaling correction. Scaled RMSEA, CFI, and TLI with Satorra (2000) scaling correction. The “Standard *χ*^2^” column contains standard test statistics, not the robust test that should be reported per model. A robust difference test is a function of two standard (not robust) statistics

*EORTC QLQ-C30* European Organisation for research and treatment of cancer quality of life questionnaire core 30, *χ*^2^ Chi-square, *df* degrees of freedom, *RMSEA r*oot mean square error of approximation, *CFI* comparative fit index, *TLI* tucker lewis index, *EORTC* European Organisation for research and treatment of cancer

*Patients with cancer who died within one year after completing the questionnaire

Releasing parameters equality constraints per item across primary cancer sites chronic lymphocytic leukemia, Non-Hodgkin lymphoma, and thyroid cancer, with reference group prostate cancer, gave us 22 models which we tested against the model with equal intercepts. We found only four out of 22 significant ∆*χ*^2^ tests (Supplement 1). Also, the changes in the item’s associated factor means were relatively small, and changed only in five cases by > 0.2. The change in factor means was associated with item SF27 in both chronic lymphocytic leukemia (0.24) and thyroid cancer (0.27), with item CF25 in both Non-Hodgkin lymphoma (0.21) and thyroid cancer (0.24), and with item CF20 in thyroid cancer (0.20). In some cases, the changes introduced by releasing item parameters partly canceled each other out on the domain level, and test scores are likely to be less biased than initially inferred [28].

When releasing item parameter equality constraints for colorectal cancer, with reference group Hodgkin lymphoma, results showed no significant *χ*^2^ tests between models (Supplement 1). Also, the changes in the item’s associated factor means were relatively small and changed only in two models by > 0.2. The change in the item’s associated factor means were associated with items CF25 (0.42) and NA14 (0.56), and canceled each other out to a certain extent on the domain level [28].

Releasing item parameters’ equality constraints for multiple myeloma, with reference group basal cell/squamous cell carcinoma showed significant *χ*^2^ tests between models in six out of 22 cases (Supplement 1). The changes in the item’s associated factor means were relatively small and changed only in one model by > 0.2. The change in the item’s associated factor means were associated with item NA14 (0.23).

### Sex-related MI

The separate CFA models showed good fit (CFI and TLI > 0.95, and RMSEA < 0.06; Table 2). When testing for MI, all models appeared to fit well (CFI and TLI > 0.95, RMSEA < 0.06) despite significant Chi-square difference tests (for equal loadings (∆*χ*^2^ *p* = 0.001) and equal intercepts (∆*χ*^2^ *p* < 0.001), Table 3). Overall, results indicate invariance of thresholds.

Releasing item parameters’ equality constraints across sex groups showed significant *χ*^2^ tests between models in 15 out of 24 cases (Supplement 1). However, the changes in the item’s associated factor means were relatively small and changed only in two models. The change in factor means were associated with item CF25 (0.26), and item NA14 (0.22). Only for the cognitive functioning did the changes in the item’s associated factor means by releasing item parameters partly canceled each other out, indicating less bias than initially inferred [28].

### Age-related MI

In the analysis for groups based on age (18–44, 45–65, and > 65 years), negative error variances for item NA14 were found and the scale Nausea/vomiting was omitted from the analysis. The separate CFA’s for the different age groups all showed good fit (CFI and TLI > 0.95 and RMSEA < 0.06, Table 2). The fit indices indicated that models with the successive constraining of threshold, loading, and intercept parameters fit the data well (Table 3; CFI and TLI > 0.95 and RMSEA < 0.06) despite significant Chi-square difference tests for the two most constrained models (∆*χ*^2^ *p* = 0.002 and ∆*χ*^2^ *p* < 0.001, respectively). Overall, results indicate invariance of thresholds.

When releasing item parameters’ equality constraints across age groups we found significant *χ*^2^ test’s between models in 10 out of 22 cases (Supplement 1). However, the changes in the item’s associated factor means were small to medium and changed only in two models for the age group 18–44 years and one model for the age group 45–65 years by > 0.2. In both age groups the change in the item’s associated factor means were associated with item CF25 (0.42 and 0.27 respectively), and in the age group 18–44 years also with item CF20 (0.39). In these cases, the changes introduced by releasing item parameters partly canceled each other out on the domain level, and test scores are likely to be less biased than initially inferred [28].

### Time since diagnosis-related MI

In the analysis of groups based on time since diagnosis (0–2, 2–5, and > 5 years), negative error variances for item NA14 were found and the scale Nausea was again omitted from analysis. The separate CFA models fitted the data well (Table 2; CFI and TLI > 0.95, RMSEA < 0.06) and Chi-square difference tests were only significant for the most constrained model (∆*χ*^2^ *p* = 0.027) (Table 3). Overall, results indicate invariance of thresholds, loadings, and intercepts across groups based on time since diagnosis. When releasing item parameters’ equality constraints we found one significant *χ*^2^ test out of 22 cases (Supplement 1), and changes in the item’s associated factor means were relatively small and all < 0.2.

### Life stage-related MI

In the analysis of groups based on life stage (i.e., cancer survivors and cancer patients in their last year of life) negative error variances for item NA14 and item CF20 were found and the scales Nausea and Cognitive functioning were excluded from further analysis. The separate CFA models showed a good fit (CFI and TLI > 0.95 and RMSEA < 0.06, Table 2). Chi-square difference tests were not significant (∆*χ*^2^ *p* > 0.05) (Table 3). Overall, results indicate invariance of thresholds, loadings, and intercepts across groups based on life stage. When releasing item parameters’ equality constraints results showed no significant *χ*^2^ test’s and changes in the item’s associated factor means were relatively small and all < 0.2 (Supplement 1).

## Discussion

As MI is necessary for valid evaluation of inter-individual differences in QoL, we tested several levels of MI of the QLQ-C30 Global QoL, functional, and multi-item symptom scales for different grouping variables using state-of-the-art multiple-group Structural Equation Modeling techniques that explicitly takes into account the ordinal measurement level of the QoL indicators. We found that the model structure fits the data well across groups, and we found empirical evidence for valid between-group comparison of QLQ-C30 latent means in subpopulations based on time since diagnosis and life stage as operationalized in our study. However, when imposing equality constraints on thresholds, loadings, and intercepts, across groups based on age, sex, and primary cancer sites results showed significantly worse fit for the model with equal loadings and the model with equal intercepts. Because there is some doubt in the appropriateness of comparing models based on goodness-of-fit indices when using DWLS for ordinal indicators [29], we also analyzed the impact of releasing thresholds and loadings for one item at a time. We found that only a few item’s associated factor means were influenced and that effect sizes were relatively small and in most cases canceled each other out.

This is in line with Costa et al. [9] who found little bias in the comparison of patients with various primary cancers. While a previous study by Marzorati et al. [8] found MI with regard to sex, we only found marginal differences on item level. Also, findings from a study by King-Kallimanis et al. [7] and Marzorati et al. [8] indicated some measurement bias based on age; however, in those studies and in our study effect sizes of the changes in the item’s associated factor means were small to medium. Other studies found measurement bias with regard to change over time (patients pre- and post-cancer treatment) [4, 5], while we, with regard to time since diagnosis, and Scott et al. 2009 [6] with regard to disease trajectory, did not. This discrepancy could be attributed to the heterogeneous cancer sample in the latter two studies, and to the operationalization of measurement occasion (i.e., within-group comparisons [7] versus between-group comparisons at various time intervals (the current study and Scott et al. 2009 [6]).

Considering the relevance of determining MI in order to legitimately compare subgroups, research into MI of questionnaires is important because both clinical and scientific decisions are based on between-group comparisons of QoL scores. QoL and other types of patient-reported outcomes are now increasingly recognized as important outcomes in cancer research, where they complement the more traditional outcomes such as overall survival [30]. Standardized questionnaires with adequate psychometric properties are also vital for daily clinical practice, as patient-reported outcomes are increasingly used to anticipate more adequately the changing problems and needs of patients [31, 32], which in turn has the potential to improve clinical outcomes (e.g., fewer emergency-room visits, fewer hospitalizations, a longer duration of palliative chemotherapy, and superior quality-adjusted survival) [31].

There are some limitations of our analyses that deserve attention. Firstly, to our knowledge, MI in the QLQ-C30 between the stages of life has not been examined previously. Our results indicate that there is no measurement bias between patients in their last year of life and cancer survivors. However, our group of patients in their last year of life was relatively small for the analysis conducted. Further analysis on this specific patient population is therefore warranted. Secondly, information about cause of death in the subgroup of patients at the end of life and to what extent these patients anticipated their death was not available in our study. It can be hypothesized that knowing that one will die in the near future may have an effect on self-reported QoL. Thirdly, our sample sizes for some of the primary cancer sites were also small. Although there is no clear guidance on sample size requirements for MGCFA with ordinal items, one of the primary cancer site groups included in the analysis barely exceeded 200. Fourth, we were not able to evaluate MI across all primary cancer types, and due to negative error variances, we had to exclude the scales Nausea and/or Pain in some of our group comparisons. We think this is because of the acuteness of symptoms like nausea/vomiting or pain, which, compared to cancer patients on active treatment, are hardly experienced in our sample that largely consisted of cancer survivors. Latest development within the EORTC Quality of Life Group has therefore been to develop a cancer survivorship assessment strategy [33]. Fifth, we were not able to control for cluster effects in our analysis because the software we used currently does not provide cluster-robust SEs/tests for ordinal data. While the intraclass correlations due to study differences between indicators of latent variables were small (< 4%), the design effect of clustering is presumably larger and leading to biased standard errors and Chi-squared tests, so the results of our study should be interpreted with care. Treating the data as continuous and using the MLR estimator while controlling for cluster effects is an alternative approach but unfortunately, we were not able to reach valid model solutions when using this approach. We note that in general, arriving at valid model estimates was challenging for the data at hand, which is presumably the result of seeking to estimate complex models with many constructs, which are each based on a limited number and highly skewed ordinal indicators and with considerably varying cluster sizes. Lastly, concerning the treatment of missing data, we have followed current EORTC guidelines. This means that our missing data approach is not a state-of-the-art method to deal with missing data. Other approaches such as Full Information Maximum Likelihood (FIML) or Multiple Imputation methods may be more appropriate and the comparison of results based on the EORTC guidelines to those acquired by different state-of-the-art imputation methods should definitely receive attention in future studies.

The strength of our study is that it is one of the largest studies into MI of the QLQ-C30, and we used a relatively large population-based sample of patients with cancer. This allowed for an extensive analysis of essential MI levels for group comparison on various patient characteristics. Our findings contribute to the methodological quality of research practices in general which have the potential to improve clinical and scientific decisions making. Our study also raises awareness about measurement bias, as this is often overlooked in the validation phase of questionnaire development [34].

In conclusion, our results show empirical evidence for the valid between-group comparison of QLQ-C30 latent means across groups of time since diagnosis and life stage. We could not confirm the highest level of MI across groups based on age, sex, and primary cancer sites. But given the few instances of non-invariance between these grouping variables, there is reason to be confident that valid conclusions can be drawn from between-group comparisons of QLQ-C30 latent means based on these characteristics. Nonetheless, future research should evaluate the potential confounding effect of variables such as treatment, age, and sex. We stress the importance of including MI evaluation in the development and validation of instruments measuring QoL in heterogeneous populations.

## Supplementary Information

Below is the link to the electronic supplementary material.

Supplementary file1 (DOCX 29 kb)

Supplementary file2 (XLSX 44 kb)

## Data Availability

The data that support the findings of this study are available from the PROFILES registry upon request.

## References

[1] Meade AW, Lautenschlager GJ (2004). A comparison of item response theory and confirmatory factor analytic methodologies for establishing measurement equivalence/invariance. Organizational Research Methods.

[2] Van de Schoot RLP, Hox J (2012). A checklist for testing measurement invariance. European Journal of Developmental Psychology.

[3] Aaronson NK, Ahmedzai S, Bergman B, Bullinger M, Cull A, Duez NJ (1993). The European organization for research and treatment of cancer QLQ-C30: A quality-of-life instrument for use in international clinical trials in oncology. Journal of the National Cancer Institute.

[4] Taminiau-Bloem EF, van Zuuren FJ, Koeneman MA, Rapkin BD, Visser MR, Koning CC (2010). A short walk is longer before radiotherapy than afterwards: A qualitative study questioning the baseline and follow-up design. Health and Quality of Life Outcomes.

[5] Gerlich C, Schuler M, Jelitte M, Neuderth S, Flentje M, Graefen M (2016). Prostate cancer patients quality of life assessments across the primary treatment trajectory: True change or response shift?. Acta Oncologica.

[6] Scott NW, Fayers PM, Aaronson NK, Bottomley A, de Graeff A, Groenvold M (2009). Differential item functioning (DIF) in the EORTC QLQ-C30: A comparison of baseline, on-treatment and off-treatment data. Quality of Life Research.

[7] King-Kallimanis BL, ter Hoeven CL, de Haes HC, Smets EM, Koning CC, Oort FJ (2012). Assessing measurement invariance of a health-related quality-of-life questionnaire in radiotherapy patients. Quality of Life Research.

[8] Marzorati C, Monzani D, Mazzocco K, Pavan F, Monturano M, Pravettoni G (2019). Dimensionality and measurement invariance of the Italian version of the EORTC QLQ-C30 in postoperative lung cancer patients. Frontiers in Psychology.

[9] Costa DS, Aaronson NK, Fayers PM, Pallant JF, Velikova G, King MT (2015). Testing the measurement invariance of the EORTC QLQ-C30 across primary cancer sites using multi-group confirmatory factor analysis. Quality of Life Research.

[10] Gotay CC, Blaine D, Haynes SN, Holup J, Pagano IS (2002). Assessment of quality of life in a multicultural cancer patient population. Psychological Assessment.

[11] Scott NW, Fayers PM, Bottomley A, Aaronson NK, de Graeff A, Groenvold M (2006). Comparing translations of the EORTC QLQ-C30 using differential item functioning analyses. Quality of Life Research.

[12] Scott NW, Fayers PM, Aaronson NK, Bottomley A, de Graeff A, Groenvold M (2007). The use of differential item functioning analyses to identify cultural differences in responses to the EORTC QLQ-C30. Quality of Life Research.

[13] van de Poll-Franse LV, Horevoorts N, van Eenbergen M, Denollet J, Roukema JA, Aaronson NK (2011). The patient reported outcomes following initial treatment and long term evaluation of survivorship registry: Scope, rationale and design of an infrastructure for the study of physical and psychosocial outcomes in cancer survivorship cohorts. European Journal of Cancer.

[14] Sangha O, Stucki G, Liang MH, Fossel AH, Katz JN (2003). The self-administered comorbidity questionnaire: A new method to assess comorbidity for clinical and health services research. Arthritis and Rheumatism.

[15] Fritz APC, Jack A (2000). International classification of diseases for oncology.

[16] Fayers PMAN, Bjordal K, Groenvold M, Curran D, Bottomley A (2001). on behalf of the EORTC Quality of Life Group. The EORTC QLQ-C30 Scoring Manual.

[17] Rosseel Y (2012). Lavaan: an R package for structural equation modeling. Journal of Statistical Software.

[18] Jorgensen TD PS, Schoemann AM, & Rosseel Y. (2020) semTools: Useful tools for structural equation modeling. R package version 0.5-3. Retrieved from https://cran.r-project.org/web/packages/semTools/semTools.pdf.

[19] Wu H, Estabrook R (2016). Identification of confirmatory factor analysis models of different levels of invariance for ordered categorical outcomes. Psychometrika.

[20] Svetina D, Rutkowski L, Rutkowski D (2020). Multiple-group invariance with categorical outcomes using updated guidelines: An illustration using Mplus and the lavaan/semTools packages. Structural Equation Modeling: A Multidisciplinary Journal..

[21] Muthén, B.O, du Toit, S.H.C., & Spisic, D. (1997). Robust inference using weighted least squares and quadratic estimating equations in latent variable modeling with categorical and continuous outcomes. Unpublished technical report. Retrieved from https://www.statmodel.com/download/Article_075.pdf.

[22] Millsap R, Yun-Tein J (2004). Assessing factorial invariance in ordered-categorical measures. Multivariate Behavioral Research.

[23] Kline RB (2016). Principles and practice of structural equation modeling.

[24] Li-tze H, Bentler PM (1999). Cutoff criteria for fit indexes in covariance structure analysis: Conventional criteria versus new alternatives. Structural Equation Modeling.

[25] Satorra A, Heijmans DDH, Pollock DSG, Satorra A (2000). Scaled and adjusted restricted tests in multi-sample analysis of moment structures. Innovations in multivariate statistical analysis: A Festschrift for Heinz Neudecker.

[26] Fischer F, Gibbons C, Coste J (2018). Measurement invariance and general population reference values of the PROMIS Profile 29 in the UK, France, and Germany. Quality of Life Research.

[27] Nye CD, Bradburn J, Olenick J, Bialko C, Drasgow F (2019). How big are my effects? Examining the magnitude of effect sizes in studies of measurement equivalence. Organizational Research Methods.

[28] Chalmers RP, Counsell A, Flora DB (2016). It might not make a big DIF. Educational and Psychological Measurement.

[29] Sass DA, Schmitt TA, Marsh HW (2014). Evaluating model fit with ordered categorical data within a measurement invariance framework: A comparison of estimators. Structural Equation Modeling A Multidisciplinary Journal.

[30] Bottomley A, Reijneveld JC, Koller M, Flechtner H, Tomaszewski KA, Greimel E (2019). Current state of quality of life and patient-reported outcomes research. European Journal of Cancer.

[31] Basch E, Deal AM, Kris MG, Scher HI, Hudis CA, Sabbatini P (2016). Symptom monitoring with patient-reported outcomes during routine cancer treatment: A randomized controlled trial. Journal of Clinical Oncology.

[32] Etkind SN, Daveson BA, Kwok W, Witt J, Bausewein C, Higginson IJ (2015). Capture, transfer, and feedback of patient-centered outcomes data in palliative care populations: Does it make a difference? A systematic review. Journal of Pain and Symptom Management.

[33] van Leeuwen M, Husson O, Alberti P, Arraras JI, Chinot OL, Costantini A (2018). Understanding the quality of life (QOL) issues in survivors of cancer: Towards the development of an EORTC QOL cancer survivorship questionnaire. Health and Quality of Life Outcomes.

[34] van Roij J, Fransen H, van de Poll-Franse L, Zijlstra M, Raijmakers N (2018). Measuring health-related quality of life in patients with advanced cancer: A systematic review of self-administered measurement instruments. Quality of Life Research.

