# Supplementary material for: Assessing measurement invariance in the EORTC QLQ-C30
Source: Qual Life Res. 2021 Jul 29;31(3):889–901. doi: 10.1007/s11136-021-02961-8 (PMC8921013; doi:10.1007/s11136-021-02961-8)
Supplement: Supplementary file 1 — Supplementary file1 (DOCX 29 kb) [file 11136_2021_2961_MOESM1_ESM.docx]

**Appendix**: Information on ethics approval for each of the studies analyzed in this manuscript

| **Study** | **Ethical board** | **Registration number** | **Date of approval** |
| --- | --- | --- | --- |
| Quality of life and health care utilization among survivors of Hodgekin Lymphoma, non-Hodgekin lymphoma and multiple myeloma | Maxima Medical Centre | METC: 0734 | 1-4-2008 |
| Life after Endometrial Cancer | Maxima Medical Centre | METC: 0733 | 2008 |
| Health-related quality of life and health care utilization in colon and rectal cancer survivors | Maxima Medical Centre | METC: 0822  CCMO: NL23463.015.08 | 15-10-2008 |
| Quality of life and health care utilization among survivors of Hodgekin Lymphoma, non-Hodgekin lymphoma and multiple myeloma | Maxima Medical Centre | METC: 0734 | 26-02-2009 |
| Quality of life and health care utilization among survivors of thyroid cancer | Maxima Medical Centre | Mei/2010 | 21-09-2010 |
| ROGYcare: the impact of cancer Survivorship Care Plan on patient reported outcomes. A pragmatic cluster randomized controlled trial | Elizabeth Hospital | METC: NL33429.008.10 | 18-10-2010 |
| Health-related quality of life and health care utilization in colon and rectal cancer survivors | Maxima Medical Centre | METC: 0822 | 17-11-2010 |
| Health-related quality of life and health care utilization in patients with prostate cancer | Catharina Hospital | 0733 | 01-08-2011 |
| Quality of life, social support and sexuality in patients with gynecological cancer and their partner | Elizabeth Hospital | METC: 1149  METC/jv/2011.129 | 06-09-2011 |
| Depressive disorders and quality of life among patients with and survivors of diffuse large-B-cell lymphoma | VU Medical Centre | METC: 2012/304 | 28-08-2012 |
| Quality of life and information provision of keratinocyte carcinoma patients | Erasmus Medical Centre | METC: MEC-2013-420 | 27-08-2013 |
| Oncology at the General Practitioner – view of the patient | Catharina Hospital | METC: 2013-64 | 30-12-2013 |
